# What Factors do Young People Define as Determinants of their Well-being? Findings from the Improve the Youth Project

**DOI:** 10.34763/jmotherandchild.2021.2503SI.d-21-00031

**Published:** 2022-03-01

**Authors:** Dorota Kleszczewska, Anna Dzielska, Agnieszka Michalska, Cátia Branquinho, Tania Gaspar, Margarida Gaspar dos Matos, Joanna Mazur

**Affiliations:** 1 Institute of Mother and Child Foundation, Warsaw, Poland; 2 Institute of Mother and Child, Institute of Mother and Child, Department of Child and Adolescent Health, Warsaw, Poland; 3 University of Warsaw, Faculty of Education, Department of Biomedical Foundations of Development and Sexology, Warsaw, Poland; 4 Institute of Environmental Health (ISAMB)/Faculty of Medicine, University of Lisbon, Lisbon Portugal; 5 Lusíada University of Lisbon, Lisbon Portugal; 6 University of Zielona Góra, Department of Humanization in Medicine and Sexology, Zielona Góra, Poland

**Keywords:** adolescents, mental health, health behaviours, well-being, life satisfaction, stress

## Abstract

**Background:**

The UN has recognised well-being as a main goal of The Global Strategy for Women’s, Children’s and Adolescents’ Health (2016–2030).

**Objective:**

The aim was to identify the areas of mental health that are the most significant to teenagers.

**Material and methods:**

The mixed-method approach was applied. Quantitative research included adolescents aged 11–15 years (6,026 in Portugal; 4,545 in Poland). HBSC study results (2013/2014) were analysed in terms of the following variables: self-rated health, life satisfaction (Cantril Ladder, KIDSCREEN-10 Index), and psychosomatic complaints (SCL scale). Focus workshops took place in 2018, with 72 teenagers aged 14 to 16.

**Results:**

16.6% of the Polish participants and 12.7% of the Portuguese participants were concerned about their health. Polish participants were less satisfied with their life [KIDSCREEN score: 25.48 for Poland (SD=6.39), and 29.96 (SD=6.03) for Portugal]. Both nations associated mental health (MH) with the family setting and relationships with friends. An additional association among Portuguese teenagers involved social issues, whereas Polish adolescents were more focussed on relationships with various people in their environment, as well as on experiencing issues at school and in the family.

**Conclusions:**

Adolescent MH is determined by stress, environmental pressure and high expectations. The viewpoints of adolescents are the most valuable source of knowledge for specialists, researchers and youth institutions, who can benefit greatly from taking advantage of this resource.

## Introduction

Adolescent well-being is deteriorating [1]. This negative trend can be observed worldwide. The United Nations has recognised this, and the well-being of adolescents became a main goal of The Global Strategy for Women’s, Children’s and Adolescents’ Health (2016– 2030) [2]. It recommends focussing on this matter actively by organising programmes, projects and campaigns, which could reverse this unfavourable tendency. In order to achieve this goal effectively, we need to define and understand the determinants of adolescent well-being. The main question that needs to be answered is how to help young people cope with issues that deteriorate their mental health (MH). According to Dunne et al. (2017) [3], diagnostic, prevention and intervention programmes are an important resource as far as adolescents are concerned, while impacting the well-being of future generations.

If we recognise that it is not easy to make young people interested in these programmes [4], one of the simplest solutions could involve asking adolescents themselves how to proceed about effecting this change. An emerging trend is to engage young people in all phases of the research [5], starting from the beginning, by giving them the opportunity to actively take part in designing the study, drawing up the questionnaire, data collection, evaluation of results, promoting the findings and implementing interventions [6]. The Improve the Youth (ItY) project was developed [4] taking into account the voice and experience of adolescents [7, 8], and is based on the premise that adolescents have the right to be included in the various endeavours that affect their lives and communities [9], and that they have the relevant knowledge concerning their own problems and needs [10].

ItY was funded by an European Union (EU) Erasmus Plus grant, and its primary aim was to support young people in one of the most crucial areas of their lives – MH. Important objectives of the project included exchanging knowledge and experience about MH problems among adolescents, as well as best practises concerning how organisations engaged in this initiative – The Institute of Mother and Child Foundation, Warsaw University, and Aventura Social – can provide young people with the best support. The aim of the project was also to convey findings from the research on adolescent MH and its determinants to adults who take care of young people daily (the medical profession, youth workers, teachers, people responsible for youth policies and young people themselves). It was also designed to provide decision-makers with relevant tools: a data report, guides and tutorials (designed by youth in cooperation with experts and practitioners), which could serve as professional aids in supporting young people in coping with stress and MH problems.

ItY was an answer to the results of the Health Behaviour of School-aged Children (HBSC/WHO) study from 2013/2014, which indicated an alarming situation regarding adolescent MH [11]. HBSC (www.hbsc.org) is a collaborative study conducted by the World Health Organization (WHO). It aims to assess health and well-being (as well as their determinants) in school-aged children in 51 countries across North America and Europe. The main goal of the HBSC/WHO study is to provide information on how to improve young people's lives. It also aims to facilitate a better understanding of health behaviours and well-being among adolescents within their social context and provides a unique opportunity for assessing subjective health and well-being among children and adolescents using multiple indicators [12, 13].

All countries participating in the HBSC study followed a standardised research protocol [12]. In Portugal, the survey has been conducted every four years since 1996 by the Aventura Social research team. Poland has been a member of the HBSC research network since 1990. The ninth national wave of the study has been carried out recently.

The HBSC study’s comparative analyses of adolescent well-being provided a foundation for further qualitative research.

### Objective

The objective of the research was to explore issues connected with MH in adolescents, identify teenagers’ perspective on MH, and establish their direct experiences in the natural context of their peer group. In summary, the aim was to identify the areas of MH that are the most significant to teenagers.

## Material and methods

The mixed-method approach was applied in the project, so the research included two parts: quantitative and qualitative methods. Quantitative research was carried out using the diagnostic survey method with the questionnaire technique.

The first part of the research included a comparative analysis of the well-being of adolescents aged 11 to 15 (6,026 in Portugal; 4,545 in Poland) [14]. The analysis aimed to examine Portuguese and Polish HBSC results from 2013/2014 concerning young people’s MH.

Statistical methods which were used in the study contained simple comparative analyses of selected MH indicators of adolescents in Poland and Portugal in the total group. Only in relation to the Kidscreen-10 index gender and age differences were included. The additional element of the analysis was an assessment of the level of social inequalities in health, based on the comparison of young people from families with different levels of affluence.

Variables referring to adolescents’ MH which were used in the first part of the research were:

Self-rated health is related to the overall assessment of one’s health status and thus connected with practices that may be regulated by efforts to achieve individual, relatively important health-related goals [15]. When asked about their general health, individuals tend to identify immediate physical cues, such as energy levels, absence of pain or recent changes in health status [16].

Life satisfaction, Cantril’s Ladder (Cantril, 1965) [17] is a single-item measure in which adolescents are asked to rate their current satisfaction with life by indicating on the ladder where on the scale they would place their lives at the moment (1 indicated the worst possible life; 10 corresponded to the best possible life). This tool has been validated in many international studies [18, 19].

KIDSCREEN-10 Index, Child and Adolescent Version. HRQoL was assessed using the KIDSCREEN-10 Index, a short version of the KIDSCREEN-52 instrument, from which 10 items were selected, consisting of a global score and a unidimensional instrument Symptom Check List (SCL). The SCL scale is a brief psychosomatic health screening tool comprising eight items and a non-clinical measure of MH, developed, validated and used in many surveys by the HBSC network [21, 22, 23].

This scale assesses the frequency of occurrence of eight subjective physical and psychological health complaints: headache, stomach-ache, backache, feeling low, irritability or bad mood, feeling nervous, sleeping difficulties and dizziness, on a five-point Likert-type scale.

The second part of the ItY project research was a qualitative study that was carried out in first quarter of 2018, where 48 teenagers form Poland and 24 from Portugal took part in the focus workshops. The teenagers were between 14 and 16 years old. The group interview method was used in the research. The discussions, based on guides prepared in advance, were moderated by a psychologist and a guidance coordinator.

During the qualitative part, adolescents had to answer to following questions:

1. How do you define mental health?
2. What areas of mental health do young people indicate?
3. Which of the indicated areas of mental health are the most important to you?
4. What factors improve, and what deteriorate, mental health in adolescence?
5. What areas of adolescent mental health need to be treated with particular attention?

## Results

### Comparative analysis of 2013/2014 HBSC findings

The results of analyses based on HBSC data from 2010 and 2014 indicated significant differences between Poland and Portugal in the subjective assessment of the health of adolescents aged 11–15 years. Usually, worse indicators were observed in the Polish sample. Adolescents’ health was defined as strong social determinant in both countries, but the level of health inequalities appeared to be higher in Portugal. Polish youth less frequently showed positive emotions, while the intensity of negative emotions was similar in both countries.

The results of the research referring to self-rated health in adolescents are presented in Table 1. The sample had an equal structure in terms of gender and age. Health, as declared by 16.6% of Polish participants and 12.7% of Portuguese respondents, is one of the adolescents’ main concerns. It has to be stressed that Polish respondents considered their health as excellent less often than Portuguese teenagers.

**Table 1:** Portuguese and Polish adolescents’ self-rated health (%)

Health	Poland	Portugal
Excellent	27.5	38.6
Good	55.9	48.7
Fair	12.7	11.7
Poor	3.9	1.0

Chi-sq=182.93, d.f. =3, p<0.001

Portuguese adolescents declared more often than Polish respondents to have excellent self-related health (38,6% vs. 27,5%). On the other hand, Polish adolescents more often answered that their self-related health is good (55,9% vs. 48,7%). Thus, 12,7% Polish respondents declared that their self-related health was fair, and it was quite similar in Portugal: 11,7%. But 3,9% Polish respondents referred to their health as poor while in Portugal, much - only 1% in comparison to Polish youth.

Portuguese and Polish adolescents declared similar life satisfaction measured by the Cantril Ladder scale (Table 2). The main differences occurred in the distribution of the answers. Polish participants got higher scores in the extreme categories of answers: 2% were extremely satisfied, and 2% were dissatisfied with their life.

**Table 2:** Polish and Portuguese adolescents’ life satisfaction according to Cantril’s ladder (%)

Life satisfaction	Poland	Portugal
Low (0-5 pct)	19.3	17.0
Medium (6-8 pct)	47.1	51.9
High (9-10 pct)	33.6	31.1
Mean ± SD	7.39±2.13	7.47±1.93

Chi-sq=21.173; d.f. =2; p<0.001; t=1.83; df = 8611; p=0.068

### What are adolescents’ psychosomatic health complaints?

Polish adolescents suffer from psychosomatic health complaints more frequently than Portuguese adolescents. About 37.2% of adolescents from Poland and 21.6% from Portugal reported two (out of eight symptoms) or more complaints occurring more than once a week. The mean complaint severity index (0–32 points) amounted to 8.91±7.76 in Poland and to 5.64±6.40 in Portugal.

All of the reported health complaints excluding backache were more common in Poland. As far as MH is concerned, adolescents from both countries reported such complaints as: feeling nervous and experiencing irritability or bad temper, with feeling dizzy being the least common complaint. All of the complaints excluding backache were more common in Poland, with the most pronounced differences visible in the case of stomachache and irritability or bad temper.

### How do teenagers define MH?

Respondents from both countries taking part in the research associated MH with the family setting and relationships with friends. Portuguese teenagers also mentioned social issues, whereas Polish ones were more focussed on relationships with various people in their environment.

Behaviour (in the negative and positive sense) was an important issue, mentioned by respondents from both countries. Teenagers from Poland and Portugal alike also linked MH with physical appearance and being satisfied with their bodies. Portuguese youths mentioned generosity and spirituality as issues connected with their MH.

A ranking of areas connected with MH mentioned by the respondents and ranked from the most to the least important can be found in Table 3. The five most significant factors reported by adolescents included stress, the family-related area, the school-related area, relationships with others and life satisfaction.

**Table 3:** Ranking of issues which Polish adolescents found as mostly connected with MH

Ranking of issues	The first group	The second group	The third group	The fourth group
1	family	future	school-related stress	relationships with others
2	stress	school	life satisfaction	stress
3	school	stress	Family	school
4	future	relationships with others	well-being	family
5	lifestyle	life satisfaction	quality of life	personality

### What do adolescents consider as their main MH determinants?

During the subsequent parts of the focus workshop, the respondents were asked to define the internal (individual, personal) and external (environmental) factors that improve or deteriorate their MH. Factors that positively affected adolescents’ well-being and life satisfaction included positive attitude to life, optimism, self-confidence, intelligence and mental resilience, as factors that facilitate maintaining MH. The primary factors that had a negative impact included sensitivity, which results in proneness to excessive worrying; shyness; pessimism and reluctance to enter social relationships.

The respondents were also asked to report external factors that can have a beneficial effect on their MH. Relationships (friends, family support, trusted people) and passions in life were mentioned the most frequently. Negative environmental determinants mentioned by young people included pressure, lack of acceptance, lack of support and examples of bullying (ridicule, bothering) among peers.

During the qualitative part of the research, the adolescents were asked to report factors concerning areas of MH that required reinforcement or improvement. Their proposals are presented in Table 4.

**Table 4:** Ranking of five most important issues that need to be improved according to adolescents

	Individual issues	Environmental issues
POLAND	1. positive attitude to life	1. friends
	2. developing an optimistic attitude	2. family support and acceptance
	3. working on one’s self-confidence	3. trusted people
	4. showing empathy	4. changing the school grading system
	5. intelligence	5. less pressure from the environment and school
PORTUGAL	1. less anxiety	1. care from healthcare professionals (psychologists)
	2. increasing trust	2. social support
	3. limited use of modern technologies	3. the need to launch new mental health measurement tools
	4. better communication with one’s loved ones	4. assets for practicing sports
	5. showing empathy	5. political honesty

## Discussion

The comparative analysis performed at the beginning of the ItY project brought a pessimistic perspective on adolescent well-being. A study developed by Feiss et al. [24] indicated that researchers' interests in adolescent well-being increases as this well-being decreases. Our study has shown that adolescents are concerned about their health, dissatisfied with their life, and suffer from psychosomatic complaints, which may be associated with cultural causes and determined by family background, school or peers [4]. Unfortunately, these reports are in line with the findings from other studies investigating the determinants of young people’s well-being, which confirms the results of first part of the ItY project [25, 26].

Adolescents participating in the study mentioned issues and problems that impact their lives and that they considered important. Furthermore, young people appreciated the opportunity to talk about their MH challenges and commended the researchers’ interest in helping them to solve those issues. They admitted that the findings would be an important source of knowledge about themselves, and the results of those discussions could be used as a means of improving their well-being.

Stress, including stress connected with school and with school education, was reported as a main MH determinant for young people from both countries. This result is consistent with the literature, which identifies school as a common stressor in adolescence [27, 28]. Answering questions about what caused this stress, they mentioned: having lots of duties, environmental pressure and high expectations that they are often unable to live up to. These findings confirm the results of the cross-sectional, school-based survey study that involved 1,816 upper-secondary school students from Mid-Norway where adolescents reported that they were stressed primarily due to interaction with teachers, peer pressure, home life, school attendance, school–leisure conflict and school itself. Researchers from Ireland who likewise engaged young people to speak about their MH problems reached similar conclusions. The young Irish also reported that high levels of stress were the most significant determinant of their well-being [29].

It can be assumed that the determinants of adolescent well-being and the stressors that they experience are similar worldwide. In Germany, in a study carried out on 350 teenagers, there was an attempt to restructure stress factors [30]. Perception of future and school were identified as the two primary sources of stress for German children, but they were also a problem for the Polish and Portuguese adolescents taking part in the ItY project. Body image, or more precisely the adolescents’ perceptions of their own bodies, also puts pressure on young people from different countries, which was already proven before in international [31] and Polish literature [32].

While analysing the determinants of adolescent MH, apart from the negative factors that affect it, it is important to understand the factors that can have a protective effect on children’s well-being. In the ITY project, the respondents mentioned support from family and friends, having trusted people around them and having passions in life, as examples of such positive factors. This is in line with findings from a Dutch study, which proved a protective effect of parent and peer support and a deleterious effect of conflictual relationships on adolescent MH. The widely known protective function of physical activity [33] and sleep duration [34] is worth mentioning in this context as well.

Nowadays, the impact that stress has on children’s well-being is even stronger, and the situation has become more severe. Young people’s MH problems have increased during the Covid-19 pandemic. In studies developed in Portugal, focussing on the voice of young people as a source of information about the effects that the pandemic had on their lives, MH emerges as a major concern during and after the pandemic [35, 36, 37]. It is safe to assume that children and adolescents were, in social terms, among the most severely affected groups as far as the isolation issue is concerned. Unexpected changes in school and social life, lack of leisure activities and limitations in contact with friends caused a severe disruption to the well-being of young people [38]. When facing this situation, it is reasonable to undertake analyses that will help determine the causes of stress in young people, how the youth themselves define these causes, and whether adolescents are able to find solutions for how to deal with these negative feelings.

## Conclusions

The suggestions for improving certain areas of MH that the young respondents mentioned are a valuable source of knowledge for young people themselves, as well as for specialists (psychologists, teacher and educators), researchers and institutions working with young people. The respondents associated elevated levels of stress with an excessive number of duties, environmental pressure and high expectations that they are often unable to live up to. Therefore, this area is an important field of research in the context of adolescent MH, and it poses new challenges for educators. The results of this article could be a valuable source also for practical implications, for example, for policy makers who create the rules of school expectations, which are remarked by adolescents as one of most stressful elements of young people’s lives.

## Ethics

The Portuguese HBSC/WHO study followed all the research guidelines outlined in the Declaration of Helsinki and has been approved by the professional Advisory Board of Equipa Aventura Social, by the Portuguese Ministry of Education, and by the scientific committee, national ethics committee (Hospital São João, Porto/São João Hospital, Oporto). Respondents provided their answers in the questionnaire anonymously; therefore, no formal approval of the National Commission of Data Protection was required. All participating schools were contacted privately, and parental informed consent was required.

The research carried out in Poland follows the HBSC international protocol as well, with considerable emphasis on the aspect of anonymity. Regional education boards (Pol. Kuratorium oświaty) are informed. Starting from the 2006 wave, the research procedure and the scope of the questionnaire have been validated by the IMC's local Bioethical Committee. At present, consent for carrying out HBSC research at particular schools has to be obtained from the schools’ Boards and Parents’ Councils. Parents sign a form expressing consent on behalf of their children (up to the age of 18) who participate in the study, whereas the students themselves express their consent by attending the study.

## Key points

1. The study was focussed on the most crucial area of adolescents’ lives, which is their MH.
2. Young people themselves have presented factors which deteriorate and improve their MH.
3. This study brings the recommendations of the directions for researchers, decision-makers and all people involved in work with youth on how to support young people in coping with their MH problems, especially with stress.
